# Physicochemical and Biological Characterization of Encapsulated Olive Leaf Extracts for Food Preservation

**DOI:** 10.3390/antibiotics12060987

**Published:** 2023-05-31

**Authors:** Wafa Medfai, Imen Oueslati, Emilie Dumas, Zina Harzalli, Christophe Viton, Ridha Mhamdi, Adem Gharsallaoui

**Affiliations:** 1 Centre of Biotechnology of Borj-Cedria, LR15CBBC05, Laboratory of Olive Biotechnology, Hammam-Lif 2050, Tunisia; 2 Faculty of Sciences of Tunis, University of Tunis El Manar, El Manar, Tunis 2092, Tunisia; 3 Univ. Lyon, University Claude Bernard Lyon 1, CNRS, LAGEPP UMR 5007, 43 Bd 11 Novembre 1918, 69622 Villeurbanne, France; 4 Univ. Lyon, University Claude Bernard Lyon 1, CNRS, IMP UMR 5223, 15 Bd André Latarjet, 69100 Villeurbanne, France

**Keywords:** olive leaf extracts, microencapsulation, encapsulating agents, spray-drying, storage stability

## Abstract

Phenolic compounds in olive leaves have an excellent antioxidant activity and good antimicrobial properties. These bioactive molecules have beneficial properties for health, arousing great scientific and commercial interest. This study reports lyophilized olive leaf extracts (OLE) encapsulated by spray-drying using maltodextrins, maltodextrins–pectin and maltodextrins–gum Arabic as encapsulating agents. Lyophilized OLE were collected from two varieties cultivated in a harsh pedo-climatic conditions of the arid region of Tunisia. The effects of the genetic factor and the different encapsulating agents on the physicochemical properties of microcapsules and their behavior during storage, as well as their antimicrobial activities, were studied. Microcapsules successfully passed heat treatment and storage conditions and their antimicrobial activities were preserved. The encapsulating agent combination improved the encapsulation efficiency and the product yield in Zarrazi variety compared to Dhokar one. In addition, Dhokar variety microparticles showed the best heat stability at 4 and 25 °C after 90 days of storage and the higher inhibition percent against bacteria. The results of the present study evidenced that the best conditions for OLE encapsulation were obtained when the maltodextrins–pectin and maltodextrins–gum Arabic were combined to form a hybrid coating material.

## 1. Introduction

Olive orchard cultivation in Mediterranean countries results in enormous amounts of by-products and residues. Olive leaves are one of these by-products. They can be found in large amounts during olive tree pruning and in the olive processing industry. The quantity of olive leaves accumulated may exceed one million tons annually in the world [1]. This by-product is characterized by high levels of polar bioactive compounds. Previous studies showed that the olive leaf is the main source of several phenolic compounds, such as oleuropein, apigenin 7-O-glucoside, luteolin 7-O-glucoside, luteolin 4′-O-glucoside, and verbascoside [2,3]. Olive leaves are increasingly attractive for health benefits [4], due to their high natural biological activity, such as antioxidant, antimicrobial [5], anti-inflammatory [6], antidiabetic [7], and antiviral effects. Additionally, they may have favorable effects in protecting against chronic diseases, including cancer [3,4,5,6,7,8], cardiovascular, and neurodegenerative pathologies [9]. It is well known that phenolic compounds also have high nutritional value, which can be applied in functional foods as natural material. Nevertheless, bioactive compounds could be significantly decreased during thermal processing and storage. In fact, the unsaturated bonds in the molecular structure of phenolic compounds, during storage, may easily lead to the degradation and deterioration of fragile components and their loss depending on external factors, such as heat, pH, exposure to oxygen, and light [1,2,3,4,5,6,7,8,9,10].

Thus, it should be interesting to find alternative techniques for solving these problems. Microencapsulation is a technology for packing or entrapping biologically active molecules (core) inside a single or complex material (microspheres), resulting in small particles or microcapsules from the submicron range to several hundred microns [11], providing protection of the compound structure, improving its stability and lengthen its shelf life against environmental conditions. Spray-drying is the most common method applied for the encapsulation of bioactive compounds in the food industry, due to its rapidity, economical processing, reproducibility, easy operation, and production of powder particles with good quality [11,12]. The choice of wall materials or encapsulating agent presents a decisive factor affecting the stability of the bioactive compounds encapsulated using spray-drying and maintaining the degree of protection of their active core [13]. The wall material serves as a physical barrier to oxygen and other environmental factors, as well as a mask for undesirable flavors and colors. Several studies demonstrated that wall materials have a strong heat-protective effect on the stability of bioactive compounds [14]. However, it is necessary to perform an appropriate selection of the encapsulating agent, since it is an interdependent operation based on the characteristics of the compound to be encapsulated [15]. Besides, the physical and chemical properties of produced spray-dried powders depend on the characteristics and structure of each encapsulating agent [16]. Maltodextrins are characterized by their film-forming ability, high water solubility, low viscosity at important concentrations, and low cost. Maltodextrins, when mixed with other coating materials, might improve the encapsulation efficiency and the stability of the microcapsules [17].

A properly combination of encapsulating agents can improve the product quality and application range of the microcapsules by compensating the defects of a single encapsulating agent [18]. In fact, maltodextrins and gum Arabic impart relatively non-hygroscopic properties to the powders made from them [19]. As a result, phenolic powders encapsulated in these wall materials became more stable during storage. Pectin is generally used in the food industry as a gelling, thickening, stabilizing, and emulsifying agent [20]. The mixture of maltodextrins and pectin could favor the protection of bioactive compounds and combines the specific properties of each polymer.

The objective of this study was to assess the microencapsulation of bioactive compounds of the OLE of two autochthonous Tunisian varieties known for their high antioxidant activities, using maltodextrins, maltodextrins–gum Arabic combinations, and maltodextrins–pectin combinations as encapsulating agents. The encapsulation of OLE was performed by the spray-drying method. The effects of several encapsulant matrices on the functional properties of microcapsules have been studied. The antimicrobial activity of microcapsules against both Gram-negative and Gram-positive bacteria was also evaluated.

## 2. Results and Discussion

### 2.1. Encapsulation Efficiency (EE)

**Figure 1:**
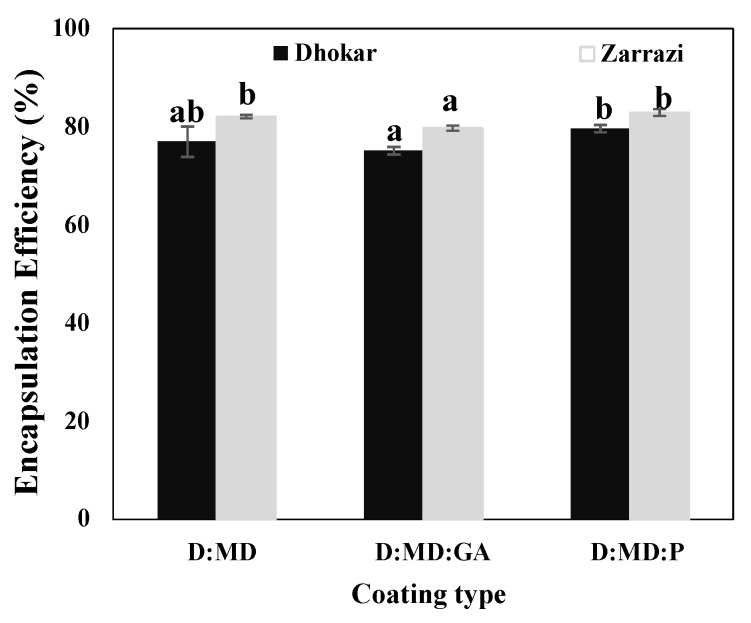
Encapsulation efficiency of spray-dried powders (Dhokar–Maltodextrins (D:MD), Dhokar–Maltodextrins–gum Arabic (D:MD:GA), Dhokar–Maltodextrins–pectin (D:MD:P), Zarrazi–Maltodextrins (Z:MD), Zarrazi–Maltodextrins–gum Arabic (Z:MD:GA), Zarrazi–Maltodextrins–pectin (Z:MD:P)). Means with the same letter are not significantly different (*p* < 0.05).

The encapsulation efficiency of the three types of microcapsules containing two varieties of OLE is presented in Figure 1. Encapsulation efficiency, representing OLE–polymer interactions, is an important parameter to encapsulate core material properly [21]. It allows to determinate the percentage of bioactive compounds of the feed, which, in fact, were encapsulated [22]. The encapsulation process is more efficient when the amount of phenolic content on the surface is smaller. In this study, as shown in Figure 1, encapsulation efficiency was between 75.0% and 80.0% for Dhokar powders, and 80.7% and 82.1% for Zarrazi powders. Our results indicated that the higher encapsulation efficiency for both varieties could be attributed to the fact that the used spray-drying method was adequate. Zarrazi variety powders with maltodextrins alone, maltodextrins–gum Arabic and maltodextrins–pectin coating material had higher efficiency than Dhokar variety powders. The higher encapsulation efficiency values in Zarrazi powders compared to Dhokar ones can be explained by the fact that phenolic compounds of each variety may be involved in different interactions with carbohydrate polymers, and/or affected by the charge, the solubility, and the molecular mobility of the phenolic compounds, as demonstrated by González-Ortega et al. [11]. Whatever the coating materials used for the two varieties, the efficiency of the microencapsulation showed little variation (Figure 1). The encapsulation efficiency can be explained by the nature of the wall material components used [23]. Indeed, the wall material can have a significant impact on the retention of phenolic compounds because it can significantly affect the patterning of the interactions between the core and the coating material [11]. Using maltodextrins alone as coating material, Zarrazi and Dhokar powders showed an encapsulation efficiency of 81.7% and 76.6%, respectively. Adding pectin to this coating material allowed to a significant (*p <* 0.05) increase on the encapsulation efficiency for Dhokar powders and maintained stable that of Zarrazi powders. Statistical analysis showed that no significant difference between maltodextrins and maltodextrins–pectin values for Zarrazi variety (*p >* 0.05). However, the lowest efficiency was obtained for microcapsules coated by maltodextrins–gum Arabic (75.0% and 80.7% for Dhokar and Zarrazi varieties, respectively) (*p* < 0.05). Consequently, maltodextrins–pectin appeared to be a clearly better carrier alternative than maltodextrins alone or maltodextrins–gum Arabic combinations in terms of encapsulation efficiency. The obtained results could be explained by the stabilizing and emulsifying effects of pectin during the encapsulation process. The same results were obtained by Sansone et al. [24], indicating that the use of maltodextrins–pectin combination as wall material for the encapsulation of *Fadogia ancylantha*, *Melissa officinalis*, and *Tussilago farfara* led to a higher encapsulation efficiency value in comparison to maltodextrins alone.

### 2.2. Drying Yields

Yield values obtained for the three formulations of Zarrazi powders ranged from 70.0% to 76.0% (Table 1), indicating relatively high yields. Remarkable differences among the different formulations of Dhokar OLE were observed, ranging from 44.3% to 74.2%. The type of the wall material strongly affected the production yields of microparticles. Among all microcapsules, the highest value was obtained for OLE of Zarrazi encapsulated with maltodextrins (Table 1). Navarro-Flores et al. [25] have reported that the difference in wall materials composition could contribute to the ability to form hydrogen bonds with polyphenols increasing or decreasing the yield. At the present experimental conditions, 75% of the polyphenols of OLE was still present in powders, suggesting that the higher inlet temperature used in this study leads to a significant positive effect on the drying yields without altering the core material [26]. The maximum value of this parameter was higher than that reported for microencapsulation of aqueous bitter melon (66.2%) [27] and salvia fruticose miller extract (60%) [28]. In the spray-drying process, losses in the recovery of microparticles was usually attributed to the deposition of the powders on the wall of the drying chamber or cyclone [22]. In fact, an accumulation of dried larger particles on the chamber wall when the drying time is longer may occur, which leads to low yields values [29]. Therefore, the production yields of microcapsules depended on the configuration of the equipment, inlet temperature, and feed flow rate used for encapsulation [30].

### 2.3. Characterization of Microcapsules

#### 2.3.1. Particle Size Distribution

The microcapsules were produced to be applied into food products. Therefore, the finesse of microcapsules and their acceptability are a critical point. In fact, the acceptability by the consumer could be widely dependent on their perception within the mouth, which is affected by their size [31]. Joye and McClements [32] have shown that particles larger than 50–100 μm can be detected as individual entities and give a gritty perception. Interestingly, in our research, the average diameter of microcapsules of Dhokar OLE with different wall materials used had a particle size ranging from 10.2 to 10.8 µm and the average diameter of microcapsules of Zarrazi powders ranged from 9.1 to 10.1 µm, which indicates the finesse of powder particles (Table 1) and the efficiency of the used method and experimental parameters. The optimized particle size obtained in the present study is due to the spraying pressure used and the high-speed homogenizer used for the dispersion of phenolic compounds into the biopolymer solutions. Similar results were found by Medina-Torres et al. [33], which showed that the particle size distribution of microencapsulated samples with suspended mucilage was less than 5 μm by the spray-drying method. Kosaraju et al. [34] have shown that the particle size distribution of chitosan-microencapsulated phenolic compounds from OLE by using spray-drying was less than 6.8 μm and the size of beads containing the extract were less than 9.9 μm. However, the particle size analysis of obtained microcapsules did not reveal a high variability depending on olive variety and wall material used although that Zarrazi microencapsulated OLE with the different wall materials exhibited slightly lower particle size than Dhokar microcapsules (Table 1).

#### 2.3.2. Scanning Electron Microscopy (SEM)

**Figure 2:**
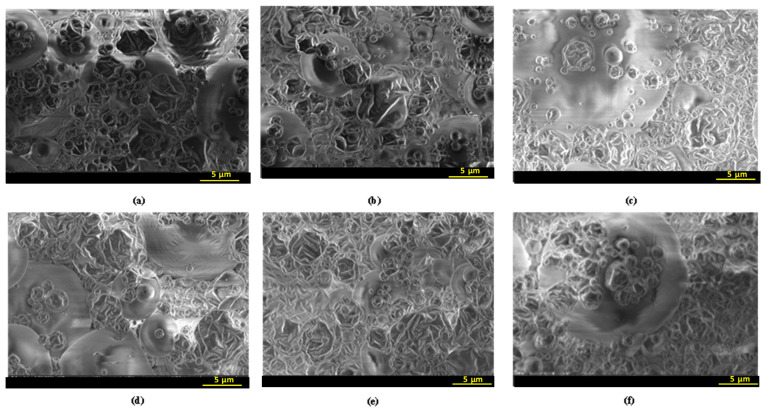
Micrograph of spray-dried powders loaded with phenolics of olive leaves extract. (**a**) Dhokar–Maltodextrins, (**b**) Dhokar–Maltodextrins–gum Arabic, (**c**) Dhokar–Maltodextrins–pectin, (**d**) Zarrazi–Maltodextrins, (**e**) Zarrazi–Maltodextrins–gum Arabic, (**f**) Zarrazi–Maltodextrins–pectin.

Scanning electron microscopy, an embedding and microtoming method, was applied to study the morphological and the inner structure of fractured microcapsules [35]. The reason for using this technique in our research is the need to determine the inner and the outer structure of the microcapsules and the encapsulating ability, and to confirm the appropriate choice of the wall biopolymers for spray-drying process. The SEM micrographs of the different microcapsules are illustrated in Figure 2. Microcapsules showed relatively rounded and spherical outer surface and irregular shape. The outer surfaces of the particles were very porous and had wrinkles with many deep dentures. According to the literature, the surface morphology and particle size of microcapsules obtained by spray-drying system are affected by various factors such as the inlet air temperature, the type and concentration of wall material, the flow velocity, and the viscosity of the feed solution [36]. Alamilla-Beltran et al. [37] found that higher air inlet temperatures (173–200 °C) resulted in more rigid microparticles and porous surfaces, as in our case (the inlet temperature used in this work was 180 °C). Medina-Torres et al. [38] mentioned that the morphological irregularities are due to the dry skins formed during removing water in the spray-drying process. In general, the brittleness of the dry skins formed by maltodextrins caused extensive cracking [35]. It is interesting to highlight that, in the present study, microcapsules with maltodextrins, maltodextrins–gum Arabic, and maltodextrins–pectin had an intact wall, without cracks (Figure 2). According to the observations of Rosenberg et al. [35], the use of gum Arabic and maltodextrins as wall polymers to encapsulate methylanthranilate or dodecane (Eastman) 15% (*w*/*w*) induced an outer surface of microcapsules with some dents and without pores or cracks. Consequently, the present results indicated the resistance of the wall constituents and their stability against the mechanical stresses during the spraying and the water removal steps and proved a good encapsulation efficiency of bioactive compounds. The outer surface of the microcapsules showed no variability with the origin of OLE, and with the different encapsulating agents, since the concentrations of gum Arabic and pectin were very low compared to the maltodextrins one (Figure 2).

### 2.4. Antiradical Activity

In order to evaluate the antioxidant activity, DPPH radical scavenging capacity of OLE was measured and results of percent inhibition are given in Table 1. DPPH radical scavenging has been widely accepted as a tool for evaluating the ability of plant extracts to scavenge free radicals generated by the DPPH reagent [39]. OLE microcapsules exhibited variable abilities to quench DPPH radical as a function of olive variety and encapsulating agent. Dhokar powders, with all encapsulating agents, exhibited the highest antioxidant activity compared to Zarrazi ones. The higher values for Dhokar powders were obtained with maltodextrins alone and the maltodextrins–gum Arabic combination, reaching a scavenging rate of 42% and 40%, respectively. Although Dhokar with maltodextrins–pectin had the highest encapsulation efficiency, it presented the lowest ability to quench DPPH. Contrary to Dhokar powders results, for Zarrazi powders, the higher values were obtained with maltodextrins–pectin combination in comparison to maltodextrins alone and maltodextrins–gum Arabic combination.

### 2.5. Heat Stability of Microcapsules

**Figure 3:**
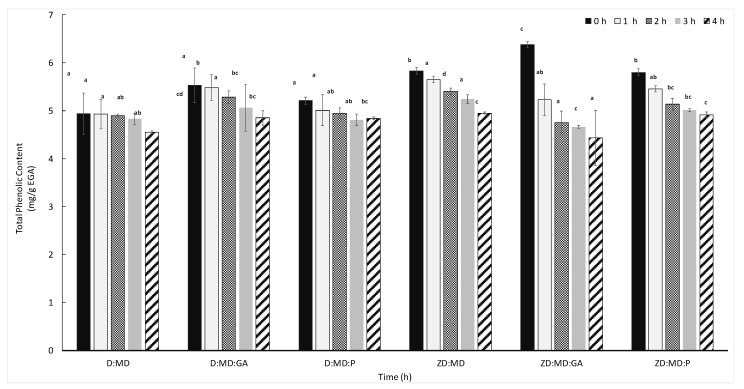
Variation of TPC values of microcapsules as a function of time at 70 °C. Means with the same letter are not significantly different (*p* < 0.05).

Thermal stability of the phenolic compounds encapsulated with maltodextrins, maltodextrins–gum Arabic combinations, and maltodextrins–pectin combinations were tested at 70 °C. The stability of the microencapsulated phenolic compounds varied according to olive variety and coating material (Figure 3). The microencapsulated phenolic compounds of Dhokar variety encapsulated in the three formulations showed a low degradation after four hours of heat treatment with an activity decrease ranging from 7% to 12%. However, a more pronounced decrease ranging from 15% to 30% was observed with the variety Zarrazi. This difference in thermal stability may be explained by the difference in the composition of the extracts between the two varieties. In our previous work [40], we showed that the phenolic compounds of the OLE of Dhokar variety were characterized by their richness in oleuropein compared to Zarrazi, which may confer a better thermal stability. According to Zoidou et al. [41], oleuropein, as a bioactive constituent added in milk, is resistant during the heating of milk and was not hydrolyzed. Maltodextrins–gum Arabic coating was the most efficient agent for the preservation of phenolic compounds against heat degradation for both Dhokar and Zarrazi powders (Figure 3). Contrary to our results, studying the microencapsulation process of phenolic compounds extracted from onion skin, Akdeniz et al. [21] demonstrated the influence of coating materials on the heat stability of microcapsules and demonstrated that casein addition to maltodextrins coating improved the heat stability compared to gum Arabic or whey protein concentrate combined with maltodextrins. The microcapsules with maltodextrins alone and maltodextrins–pectin showed a better preservation for the Dhokar than Zarrazi variety.

### 2.6. Storage Stability

**Figure 4:**
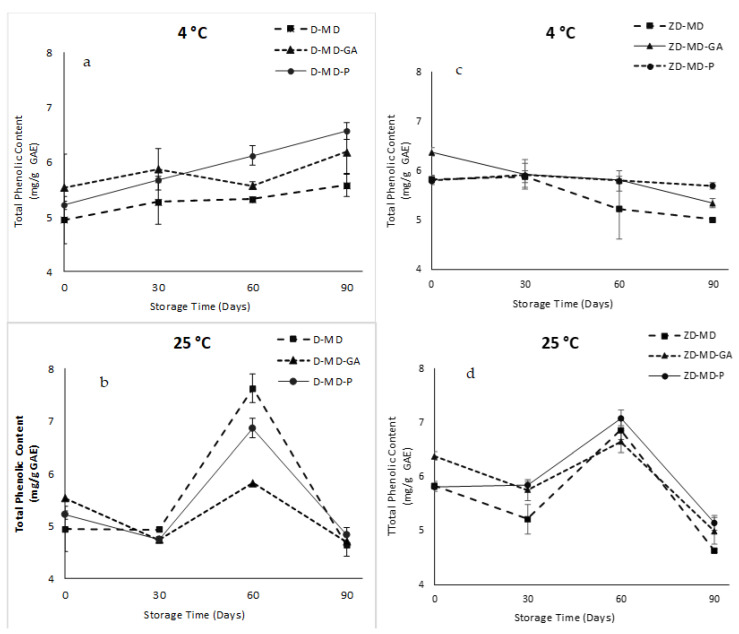
Storage stability of microcapsules at 4 and 25 °C for 90 days. (**a**) Dhokar microcapsules stored at 4 ° C, (**b**) Dhokar microcapsules stored at 25 ° C, (**c**) Zarrazi microcapsules stored at 4 °C, (**d**) Zarrazi microcapsules stored at 25 °C.

In order to evaluate the storage stability of OLE powders, microcapsules were stored at 4 °C and 25 °C during 90 days. The obtained results are summarized in Figure 4. Storage of the microencapsulated OLE with different coating materials showed a remarkable increase in the total phenolic content of the Dhokar variety at 4 °C (Figure 4a). The highest increase in total phenolic compounds was observed in maltodextrins–pectin encapsulating agent. This increase may be a result of the polyphenols recovery and formation during storage time. Similar behaviors were observed by Bakowska-Barczk and Kolodziejczyk, [42], Nunes et al. [43], and Saénz et al. [44], who found recoveries and formation of polyphenols as a consequence of the hydrolysis of concentrated *Ilex paraguariensis* extract, cactus pear, and black currant polyphenol conjugates, respectively, leading to an increase of microcapsules phenolic compounds after 12 months, 45, and 44 days of storage, respectively. The phenolic extract of the Zarrazi powders microencapsulated with maltodextrins and maltodextrins–gum Arabic combination decreased gradually during storage at 4 °C (Figure 4c), whereas it remains constant when using maltodextrins–pectin coating materials. After storage at 25 °C, the two varieties of OLE with different encapsulating agents showed a significant increase, after 60 days of storage, followed by a significant decrease at the end of the experimentation (Figure 4b,d). Zarrazi powders with maltodextrins–gum Arabic and maltodextrins alone as encapsulating agents showed the highest degradation rate for polyphenols compared to pectin during storage at 25 °C after 90 days. Zarrazi powders with maltodextrins–pectin showed a slightly degradation (Figure 4d). Consequently, Zarrazi powders with maltodextrins–pectin seems to be the most stable coating materials during storage and showed a significantly higher protective effect than maltodextrins alone and the maltodextrins–gum Arabic combination. These results show the importance of the encapsulating material in the stability of bioactive compounds on the one hand, and the correlation between the stability of phenolic powders and the encapsulation efficiency of microparticles on the other hand. The higher efficiency of the microencapsulation process can be correlated to the higher stability of the powders. This observation is similar to that made by Yinbin et al. [14]. Therefore, microencapsulation by the spray-drying method could successfully diminish the damage of phenolic compounds caused by several environmental conditions [14]. In fact, the storage of microencapsulated bioactive compounds at a low temperature could minimize or improve the long-time heat stress of both the encapsulating agent and active compounds [44,45].

### 2.7. Determination of Antimicrobial Activity of Microencapsulated OLE

**Figure 5:**
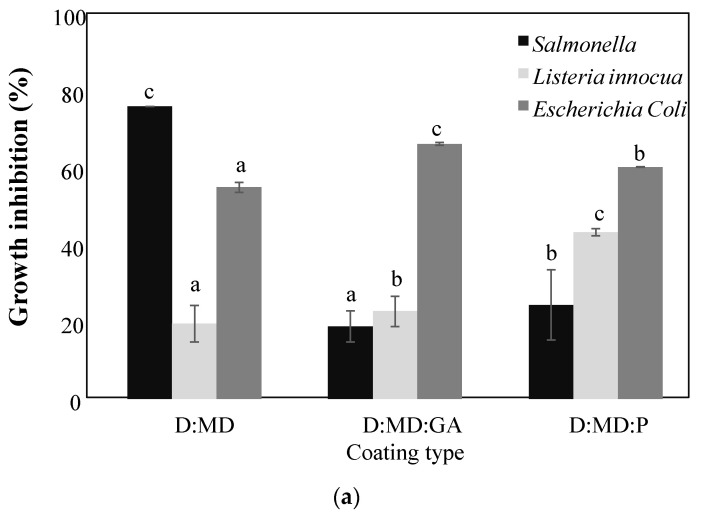

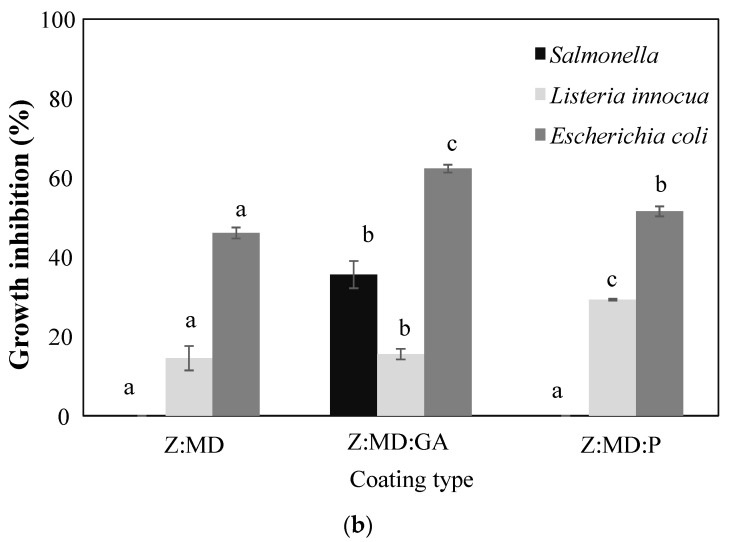
Growth inhibition percentage of six types of spray-dried powders, i.e., Dhokar OLE (**a**) or Zarrazi OLE (**b**) with maltodextrins alone, Maltodextrins–gum Arabic, or Maltodextrins–pectin combinations, tested at 5 mg/mL against three bacterial strains, i.e., *Salmonella enterica*, *Listeria innocua*, and *Escherichia coli*. Different letters indicate significant differences in the means (*p* < 0.05).

OLE are receiving great interest due to their well-known antimicrobial activity. The antimicrobial properties of OLE against different pathogen microorganisms were reported by several researchers [2,3,4,5]. In the present study, the antimicrobial activities of microencapsulated OLEs against Gram-negative (*E. coli* and *S. enterica*) and Gram-positive (*L. innocua*) bacteria are given in Figure 5. The data revealed that OLE powders of Dhokar and Zarrazi varieties showed an inhibition effect on the growth of all examined bacteria species at 5 mg/mL concentration, indicating interesting antimicrobial activities. The obtained results are in agreement with the observations of Bayraktar et al. [46], who found that OLE-loaded fibroin microparticles formed by spray-drying dropped the absorbance values against *E. coli* (NRRLB-3008) and *Staphylococcus epidermidis* (ATCC 12228) at 600 nm at a concentration of 10 mg/mL. A significant variation was detected in the percentage of bacteria inhibition between the studied OLE varieties and between encapsulating agent types. In fact, microcapsules of Dhokar OLE exhibited higher inhibition effects by comparison to the microcapsules of Zarrazi OLE for *S. enterica*, *E. coli*, and *L. innocua* bacteria (*p* < 0.05). *S. enterica*, a Gram-negative bacterium, was the most sensitive to Dhokar microencapsulated OLE compared to *E. coli* and *L. innocua*, with maltodextrins as encapsulating agent (Figure 5a). However, Zarrazi variety had no inhibition effect on *S. enterica* using maltodextrins alone and maltodextrins–pectin as encapsulating agents (Figure 5b). Generally, Gram-negative bacteria are more resistant than Gram-positive bacteria due to the structural characteristics of the bacteria [5]. Indeed, Gram-negative cells have an extra outer membrane, which is not the case for Gram-positive bacteria [47]. The use of the maltodextrins–pectin combination increased by two-fold the percentage of inhibition growth of *L. innocua* compared to maltodextrins and maltodextrins–gum Arabic for both varieties with higher values for Dhokar OLE (Figure 5a,b). Interestingly, powders with the maltodextrins–gum Arabic combination increased the inhibition effect of *E. coli* compared to maltodextrins–pectin and maltodextrins alone for both varieties, with higher values for Dhokar OLE (Figure 5a,b). The present study showed that the inhibition of bacteria was closely related to the type of encapsulating agent, with the most interesting results when encapsulating agents are combined.

## 3. Materials and Methods

### 3.1. Chemicals, Reagents, and Bacteria

Maltodextrins DE 12 (dextrose equivalent value of 12) were obtained from Roquette-freres SA, (Lestrem, France), while gum Arabic, pectin, sodium carbonate (Na_2_CO_3_), gallic acid, Folin Ciocalteu, 2,2-diphényl 1-picrylhydrazyle (DPPH), and ethanol were purchased from Sigma-Aldrich Chimie (St. Quentin Fallavier, France). The target strains used in this study were *Listeria innocua* strain DSM 20649, *Salmonella enterica* serovar typhimurium strain DSM 11320, and *Escherichia coli* strain DSM 613 from DSMZ German Collection of Microorganisms. All stains were maintained at −20 °C in Tryptone Soy Broth (TSB, Biokar Diagnistics, Allonne, France) supplemented with 20% (*v*/*v*) glycerol.

### 3.2. Sampling

The region of Tataouine in Tunisia is characterized by an extremely high temperature, low rainfall, and soil erosion. All of the mature leaves used in this study were collected from olive trees in January 2020 that grew on traditional rain-fed, high-altitude farms. Both varieties are characterized by good oil quality, high stability, and being rich in antioxidants [48].

### 3.3. Preparation of Olive Leaf Extracts

Leaves were randomly harvested from different parts of the trees and immediately transferred to the laboratory to be lyophilized after washing with distilled water. Then, they were ground to fine particles prior to extraction. A sample of 200 g of olive leaves from each variety was mixed with 2 L of 47% ethanol. Hydrophilic compounds from olive leaves were extracted using an ultrasonic bath (40 kHz) (Ultrasons JP., Selecta, Barcelona, Spain) for 50 min at room temperature (25 °C). The solution was filtered with a Whatman No. 1 paper and then concentrated on a rotary evaporator (Evaporator R100., Büchi, Switzerland) at 38 °C until its volume decreased to 2/3rd of the initial value. The samples were freeze-dried, and the concentrated extracts were frozen for 1 day. The resulting dried OLE were stored at −20 °C in dark bottles.

### 3.4. Preparation of the Microcapsules

Three kinds of encapsulating agents were used including maltodextrins, maltodextrins–gum Arabic, and maltodextrins–pectin combinations. Encapsulation was prepared as follows: for 100 g of solution, OLE (1% *w*/*w*) was dissolved in distilled water at room temperature (25 ± 1 °C) until a homogeneous solution was obtained, and then mixed with a maltodextrins solution alone (20%) under constant stirring, or adding gum Arabic (0.25%) or pectin (0.25%). The mixture was homogenized with a magnetic stirrer (300 rpm) for 1 h at 25 °C. Dispersions were then spray-dried using a Mini Spray-Dryer (Büchi B-290, Switzerland). The values of operating parameters established for the drying process were: solid content 10 g/100 g, inlet air temperature 180 °C, outlet air temperature 80 ± 3 °C, and feed flow rate 500 mL/h. The spray nozzle diameter was 0.5 mm. The same parameters were used for all formulations. The spray-dried powders obtained were stored in the dark at −20 °C for further evaluation.

### 3.5. Encapsulation Efficiency (EE) and Load Yield (Y)

Encapsulation efficiency has been determined by adapting the method and equations presented by Laine et al. [49] on the microencapsulated products. An amount of each powder (17 mg) was weighted on microcentrifuge tubes, to which 1.4 mL absolute ethanol was added. Powder suspension was immediately vortexed (10 s) and centrifuged at 12,000× *g* for 2 min at room temperature (25 °C). The supernatant ethanol fraction was collected and the pellet was dissolved in 1.4 mL of water by vortexing. Both fractions were analyzed for Total Phenolic Content (TPC) and antioxidant capacity. Phenolic compounds in the ethanol fraction were considered to be surface/non-encapsulated fraction, while phenolics in the water fraction corresponded to the encapsulated fraction. Phenolic content measurements (Section 3.6) were performed using the Folin–Ciocalteau method [50]. The encapsulation efficiency (*EE*) for total phenolics was calculated according to Equation (1):

(1) EE%=Encapsulated phenolics mggEncapsulated phenolics mgg+Surface phenolics mgg×100

The microencapsulating yield (Y) was determined gravimetrically according to Equation (2). This parameter corresponds to the ratio of the mass of the powder obtained at the end of the process in the vessel to the mass of the initial substances (solids of OLE extracts and encapsulants) in the feed suspensions:

(2) $$Y\;\left(\%\right)=\frac{\mathrm{Powder}\;\mathrm{after}\;\mathrm{Spray}\;\mathrm{Drying}\;\left(g\right)}{\;\mathrm{Solids}\;\mathrm{in}\;\mathrm{the}\;\mathrm{feed}\;\mathrm{solution}\;\left(g\right)}\times 100$$

### 3.6. Total Phenolic Content (TPC)

The TPC of OLE powders was evaluated by using the Folin–Ciocalteau reagent [50]. Solutions with resuspended microencapsulated powders (125 µL) were diluted with deionized water to a volume of 500 µL, 125 µL Folin–Ciocalteu reagent (Sigma-Aldrich) was added, and the solution was then incubated for 3 min in the dark before adding 1250 µL of a 7% (*w*/*v*) Na_2_CO_3_ solution. Then, deionized water was added to a final volume of 3 mL. The solution was kept in the dark at room temperature for 90 min and TPC was determined by reading the absorbance at 765 nm using a spectrophotometer (UV-3100PC Spectrophotometer). Results were expressed as mg of gallic acid equivalents g^−1^ of dry weight (GAE g^−1^ DW) of sample. Each sample was analyzed in triplicate.

### 3.7. Antioxidant Capacity (DPPH Radical Scavenging Method)

The DPPH radical-scavenging activity of microencapsulated OLEs was determined by adapting the protocol described by Bersuder et al. [51] with some modifications. An aliquot of 500 µL of each microencapsulated OLE was mixed with 375 µL of ethanol and 125 µL of a daily prepared solution of DPPH (0.02% *w*/*v* in ethanol). The mixtures were shaken and then incubated for 60 min in dark at room temperature. The reduction of DPPH radical was measured at 517 nm.

DPPH radical scavenging activity (%) = ((A_control_ − A_sample_)/A_control_) × 100

A_control_: the absorbance of DPPH radical + solvent;

A_sample_: the absorbance of DPPH radical + sample.

### 3.8. Physical Properties of Microcapsules

#### 3.8.1. Particle Size Distribution

Particle size distribution of spray-dried powders were assessed by a laser diffraction instrument (Mastersizer 3000, Malvern Instruments, Malvern, UK) with a value of 1.5 for the relative refractive index (droplet to solvent), 0.1 absorption, and an index of refraction of 1.36 for ethanol. For measuring the particle size distribution of spray-dried capsules, the powder was slowly added into the measurement chamber containing ethanol. The spray-dried powders were continuously stirred throughout the measurement to ensure that the samples were homogeneous. The volume mean particle diameter (D_43_) was calculated by the software from the three injections of three separate samples with five readings per sample.

#### 3.8.2. Particle Morphology

Morphological characteristics of the microencapsulated particles were evaluated using a scanning electron microscope (Quattro S., ThermoFisher, Waltham, MA, USA).

### 3.9. Storage Stability of Microcapsules

Three types of microencapsulated OLE powders were sealed in aluminum foil bags, and then stored in an incubator at 4 and 25 °C for 90 days. The total phenol retention of the samples was determined every 30 days during storage. Phenolic content measurements were performed using the Folin–Ciocalteau method [50].

### 3.10. Heat Stability Test of Microcapsules

The heat stability of microcapsules was evaluated with regard to phenolic compounds stability during storage at 70 ± 1 °C for 4 h. Samples were placed in amber vials. To determine the total phenolic content (TPC), duplicate vials were removed every 1 h until assay was completed. Phenolic content measurements were performed using the Folin–Ciocalteau method [50].

### 3.11. Determination of Antimicrobial Activity

The antimicrobial activity was determined against a Gram-positive bacterium, *Listeria innocua* strain DSM20649, and two Gram-negative bacteria, *Escherichia coli* strain DSM613 and *Salmonella enterica* strain DSM11320. Strains were stored at −20 °C in Tryptone Soy Broth (TSB) (Biokar diagnostics, Beauvais, France) with 15% (*v*/*v*) of glycerol. One milliliter of the stock culture was transferred to 9 mL of TSB and incubated for 8 h at 30 °C. One milliliter of this pre-culture was then transferred in 9 mL of TSB and incubated overnight at 30 °C. OLE powders were dissolved in TSB to prepare a solution at 5 mg/mL. In a 96-well plate, 30 μL of bacterial suspension at 10^6^ CFU/mL were added in wells containing OLE solution in TSB at a 5 mg/mL concentration. Each test plate included growth controls (bacteria at 5 × 10^6^ CFU/mL in TSB without microcapsules) and sterility controls (TSB alone or OLE powders in TSB, without bacteria). All tests were performed in triplicates. The plates were incubated at 30 °C under shaking in a Multiskan SkyHigh equipment (Thermofisher scientific), which measured the OD_600_ every 15 min during 24 h. The OD_600_ of sterility control was subtracted to the OD_600_ of growth curves. The mean OD_600_ nm values were plotted versus time. The growth inhibition (*GI*) was determined according to Equation (3):

(3) $$GI\;\left(\%\right)=\left(\frac{OD\;growth\;positive\;control-OD\;growth\;with\;extract}{OD\;growth\;positive\;control}\right)\times 100$$

### 3.12. Statistical Analyses

All the experiments were performed in triplicate. The results were reported as mean values of three replicates ± the standard deviation (SD). The significance of differences among mean values was determined by one-way ANOVA. Comparisons among means were performed using Duncan’s multiple range test. The differences between the average variables were considered significant at *p <* 0.05. The statistical analysis was performed using SPSS 16.0 (SPSS Inc., 2007).

## 4. Conclusions

In this study, Dhokar and Zarrazi OLE were coated with different coating materials in order to choose the variety with the better encapsulation formulation in terms of biological activity. Between the two varieties, Zarrazi OLE showed the highest encapsulation efficiency values and the best drying yields with different encapsulation agents. Dhokar powders with different encapsulating agents were effective in scavenging radicals when assessed by DPPH assay and showed the more homogeneous particle size distribution. The addition of pectin to the coating material reduced the loss of phenolics for Zarrazi variety during storage at 4 and 25 °C and that of Dhokar variety at 4 °C. The microcapsules of both varieties were stable during storage at 70 °C for 4 h. The most phenolic stability was observed when the maltodextrins–gum Arabic combination was used as encapsulating agent. In addition, by using the maltodextrins–gum Arabic coating material combination, a significant percentage of inhibition against *E. coli* and maltodextrins–pectin provided higher percentage of inhibition growth of *L. innocua*, for both varieties, with higher values for Dhokar OLE. Consequently, the most important result of this research is the relationship between the encapsulating agents and the stability of phenolic compounds. The spray-drying microencapsulation was shown to be an effective way of stabilizing phenolic compounds and ensuring a longer shelf life of food products when these microcapsules will be used as food preservatives.

## Figures and Tables

**Table 1:** Drying yields, particle size, and antiradical activity values of microcapsules having different coating materials. Different letters indicate significant differences in the means (*p* < 0.05).

Coating Type	Drying Yield (%)	Particle Size (µm)	Antiradical Activity (%)
Dhokar–Maltodextrins	44.3 ± 1.72 ^a^	10.2 ± 0.37 ^c^	42.05 ± 0.01 ^d^
Dhokar–Maltodextrins–gum Arabic	74.2 ± 2.36 ^d^	10.3 ± 0.27 ^c^	39.95 ± 0.02 ^d^
Dhokar–Maltodextrins–pectin	68.5 ± 1.89 ^b^	10.8 ± 0.21 ^d^	31.07 ± 0.01 ^c^
Zarrazi–Maltodextrins	76.0 ± 2.02 ^d^	10.1 ± 0.23 ^b^	20.39 ± 0.01 ^a^
Zarrazi–Maltodextrins–gum Arabic	71.9 ± 1.58 ^c^	09.8 ± 0.29 ^b^	22.05 ± 0.02 ^a^
Zarrazi–Maltodextrins–pectin	70.0 ± 2.17 ^c^	09.1 ± 0.27 ^a^	25.37 ± 0.03 ^b^

## Data Availability

Not applicable.
